# The value of hepatic diffusion-weighted MR imaging in demonstrating hepatic congestion secondary to pulmonary hypertension

**DOI:** 10.1186/1476-7120-8-28

**Published:** 2010-07-21

**Authors:** Yuksel Dogan, Aliye Soylu, Ozgur Kilickesmez, Tuna Demirtas, Kadriye Orta Kilickesmez, Sebahat Nacar Dogan, Gulay Eren, Isa Sevindir, Nurgul Yasar, Sule Poturoglu, Kenan Sonmez

**Affiliations:** 1 Department of Cardiology, Bakirkoy Dr. Sadi Konuk Education and Research Hospital, Istanbul, Turkey; 2 Department of Gastroenterology, Bakirkoy Dr. Sadi Konuk Education and Research Hospital, Istanbul, Turkey; 3 Department of Radiology, Yeditepe University, Istanbul, Turkey; 4 Department of Radiology, Bakirkoy Dr. Sadi Konuk Education and Research Hospital, Istanbul, Turkey; 5 Department of Cardiology, Institute of Cardiology, Istanbul University, Istanbul, Turkey; 6 Department of Radiology, Taksim Education and Research Hospital, Istanbul, Turkey; 7 Intensive Care Unit, Bakirkoy Dr. Sadi Konuk Education and Research Hospital, Istanbul, Turkey; 8 Department of Internal Medicine, Bakirkoy Dr. Sadi Konuk Education and Research Hospital, Istanbul, Turkey; 9 Department of Gastroenterology, Haseki Education and Research Hospital, Istanbul, Turkey; 10 Department of Cardiology, Kosuyolu Heart and Research Hospital, Cardiology Clinic, Istanbul, Turkey

## Abstract

**Background:**

Congestive hepatomegaly might be the first sign for pulmonary hypertension. Apparent diffusion coefficient (ADC) value obtained with quantitative diffusion-weighted magnetic resonance imaging (DW-MRI) is affected by liver fibrosis and perfusion. We aimed to evaluate the diagnostic value of DW-MRI in cooperation with biochemical markers, ultrasonography (US) and echocardiography (TTE) in determining the degree of hepatic congestion secondary to pulmonary hypertension (PHT).

**Methods:**

35 patients with PHT and 26 control subjects were included in the study. PHT was diagnosed if pulmonary artery systolic pressure (PASP) was measured above 35 mmHg with TTE. Study group was classified into mild and moderate PHT. DW-MRI was performed with b-factors of 0, 500 and 1000 sec/mm². Mean ADC, ADC-II (Average of the ADC values of right lobe anterior and posterior segments), US, TTE and blood biochemical parameters of both groups were compared.

**Results:**

There exists a positive correlation between liver size and the diameters of vena cava inferior, right atrium, right hepatic vein(RHV), mid-hepatic vein(MHV), left hepatic vein(LHV) (p < 0.01). There was a positive correlation between PASP and RHV, MHV, LHV. The patients had lower ejection fractions (p < 0.01) and higher LDH (p < 0.01) and ALP (p < 0.05) levels than the control group. The ADC values of the patients with moderate PASP were higher than those with a mild PASP (p < 0.05). Mean ADC was higher in patients with moderate PHT compared to control group (p = 0.009). There was a positive correlation between PASP and ADC values of right lobe posterior segment of the liver (p < 0.05). The ADC-II and mean ADC values of the patients with moderate PASP were higher than those of the control group (p < 0.01).

**Conclusions:**

Congestion due to moderate PHT might be diagnosed with DW-MRI. As PASP increase; mean ADC and ADC-II values increase.

## Introduction

Pulmonary hypertension (PHT) is a disease caused by various etiologies which may lead to right heart failure and death. Pulmonary artery pressure and pulmonary vascular resistance increases due to the physiopathological and histopathological changes (vasoconstriction, vascular proliferation, *in situ *thrombosis and vascular remodeling) observed in PHT [1,2]. Disease may result in centrilobular necrosis due to chronic passive liver congestion if left untreated and present with edema, ascites, jaundice, elevated biochemical markers and even death [1-4]. Transthoracic Doppler echocardiography (TTE) is a non-invasive test used for cardiac evaluation to determine pulmonary arterial systolic pressure in the presence of tricuspid failure (sensitivity 100%, specificity 96%) [5,6].

Enlarged liver in addition to dilatation of hepatic veins and inferior vena cava (IVC), both of which may be demonstrated with ultrasonography (US), may result from hepatic congestion. However, these signs are observed in advanced PHT cases and hepatomegaly plus intra-abdominal free fluid may be noted with increased severity [7,8]. Signs of liver congestion due to early PHT might be important in clinical differential diagnosis of hepatomegaly.

There are several publications indicating the efficacy of quantitative apparent diffusion coefficient (ADC) measurement with diffusion weighted magnetic resonance imaging (DW-MRI) in proving liver fibrosis and evaluating other space occupying lesions [9]. Diffusion weighted imaging is an advanced application of MRI used in evaluating the microscopic structure of tissues. This imaging method relies on quantification of the diffusion of water molecules inside tissues. Combined with other methods, this imaging modality might be used in evaluating parenchymal tissue that has no proven abnormalities with routine imaging modalities [10].

In our prospective study, we aimed to evaluate the diagnostic value of DW-MRI in cooperation with biochemical markers, US and TTE in determining the degree of hepatic congestion secondary to PHT.

## Materials and Methods

The study was performed on 61 subjects, consisting of 39 (63.9%) females and 22 (36.1%) males with an age range of 25 to 82, recruited between July 2008 and February 2009. Mean age of the subjects was 53.51 ± 13.98. Written informed consent was obtained from all subjects in conformity with the requirements of local ethics committee. Subjects were divided into two groups as "study group" (n = 35) and "control group" (n = 26). Study group was composed of patients diagnosed with TTE confirmed secondary PHT with underlying diseases such as rheumatic heart disease, chronic obstructive lung disease and congenital heart disease in the etiology. Patients with normal TTE findings were included in the control group. Lack of right heart failure, another systemic disorder or malignity, chronic liver disease and hepatic vascular pathology, acute and chronic hepatic diseases (viral, autoimmune markers, infectious, infiltrative, TSH, FT4, AST, ALT, total bilirubin, indirect bilirubin, LDH, total protein, albumin) as revealed by the past medical history, physical examination and laboratory findings were the inclusion criteria of the study applied for both groups. A diagnosis of PHT was established according to pulmonary arterial systolic pressure (PASP) > 35 mmHg recorded by TTE. Diagnosis of PHT was based on from the European Society of Cardıology and the European Respiratory Society Guidelines for the Diagnosis and Treatment of PHT [11]. Patients with PHT were grouped as mild PHT (35-50 mmHg) and moderate PHT (51-70 mmHg) according to their PASP. Patients with the diagnosis of secondary PHT were included in the study. Twenty six patients had mild PHT, whereas 9 had moderate PHT.

The physical examination and evaluation of the patients was performed by a cardiologist and gastroenterologist independently. The history of the patients were particularly assessed concerning orthopnea, paroxysmal nocturnal dyspnea (PND) and effort dyspnea. During cardiac evaluation all minor and major criteria of heart failure like jugular venous pressure (JVP), jugular-venous reflex (JVR), hepatomegaly, and pretibial edema were evaluated. Patients with high JVP, positive JVR, orthopneic and PND patients, patients with congestive and right heart failure or patients under treatment, moderate tricuspid regurgitation and finally patients with critical PHT were excluded from the study. 

All patients had a full history taken for alcohol use, medications and risk factors for viral hepatitis. Electrocardiography, chest X-ray, echocardiography, aspartat transaminase (AST), alanine transaminase (ALT), lactate dehydrogenase (LDH), alkaline phosphatase (ALP), total and indirect bilirubin, upper abdominal US and liver DW-MRI were performed in all patients and control group. All patients had afull history taken for alcohol use, medications and risk factors for viral hepatitis.

Patients were excluded from the study, if they had acute coronary syndromes, severe functional capacity (NYHA-IV), a diagnosis of or a symptom suggesting chronic liver disease, malignity, any contraindication for MRI, inadequate echocardiographic evaluation, severe PHT and patients with severe tricuspid regurgitation.

Two-dimensional continous doppler, color doppler recordings of patients via TTE (GE, Vivid S5, Norway) with a 2.5 - 3.5 MHz phased-array transducer were provided by an experienced cardiologist, who had no information about the patient's medical history, liver function test, US and MRI findings. TTE was used to measure left ventricular ejection fraction (EF), diameters of right ventricle (RV), right atrium (RA), left heart chambers and tricuspid failure [12]. The diameter of RV and mediolateral diameter of midcavitary RA were calculated in apical four-chamber position, in the end. The heart chambers with the following measurements (RA > 45 mm, LA > 40 mm, RV > 28 mm) were accepted as enlarged [13]. Right ventricular systolic pressure was calculated from tricuspid failure in apical four chamber position, according to Bernoulli equation: RVSP (mmHg) = 4x (V^2^) + right atrial pressure) [1], (Table 1). RA pressure was estimated according to the size of the inferior vena cava and the changes in its size during respiration (a value of 5 mm Hg was assigned to a small vena cava collapsing 50% in diameter during quiet inspiration, a value of 20 mm Hg was assigned to a very dilated vena cava without respiratory variation in size, and values of 10 to 15 mm Hg were assigned to intermediate findings) [13].

**Table 1:** Estimation of right atrial pressure

IVC-diameter- cm	Respiratory Motion%	mRAP(mmHg)
<1.5	100	<5

1.5 - 2.5	>50	5-10

1.5 - 2.5	<50	10-15

>2.5	>50	15-20

2.5 + dilated	0	>20

*Abb*: *IVC*: Inferior vena cava, *mRAP*: mean right atrial pressure

### Ultrasonography

Abdominal US evaluations of the patient and control groups were performed in supine position subsequent to 6 hours of fasting. GE Logic 9 device (GE Medical Systems, Milwaukee, USA) with 3.5-5 mHz convex probe was used for the evaluations with two accompanying radiologists with expertise on abdominal radiology. Craniocaudal (longitudinal length) measurements of the liver were performed in the mid-clavicular axis. Diameter of IVC was measured 1 cm above the merging point of hepatic veins, right hepatic vein (RHV), left hepatic vein (LHV), and middle hepatic veins (MHV) were measured from 1 cm to IVC, and portal vein (PV) was measured at the level of hilus of the liver. Liver parenchymal echogenicity was found to be homogenous and normal in all subjects (study and control groups) and space occupying lesions were neglected including cysts and hemangiomas that were smaller than 1 cm.

### MR Imaging

MR imaging was performed on a 1,5 T body scanner (Avanto; Siemens, Erlangen, Germany) with a 33 mT/m maximum gradient capability using an eight channel phased-array body coil.

Before diffusion weighted imaging, breath hold, axial 3D gradient-echo T1-weighted sequence; (repetition time [TR], 5.32 ms; echo time [TE], 2.58 ms); 2D gradient-echo T1 in-phase and out-of-phase (TR, 128 msec; in-phase TE, 4.89 msec; out-of-phase TE, 2.38 msec), axial respiratory-triggered, turbo spin-echo T2-weighted sequence with fat saturation (TR, 1900 ms; TE, 76 ms), coronal T2-weighted half-Fourier single-shot turbo spin-echo (HASTE) (TR, 1100 ms; TE, 116 ms) sequences and then diffusion weighted single-shot spin-echo echo-planar sequence with, chemical shift selective fat-suppression technique; TR/TE, 4900/93; matrix, 192 × 192; slice numbers, 30; slice thickness = 6 mm; interslice gap, 35%; FOV, 45 cm; averages, 5; acquisition time, approximately 3 minutes, PAT factor, 2; PAT mode, parallel imaging with modified sensitivity encoding (m SENSE) was performed. DW-MRI was performed with b-factors of 0, 500 and 1000 sec/mm².

### Image interpretation

The DWI datasets were transferred to an independent Workstation (Leonardo console, software version 2.0; Siemens) for postprocessing, and the ADC maps were reconstructed.

To measure ADC value two abdominal radiologists established round regions of interest (ROI) on the right lobe posterior (RLP) and anterior (RLA) and left lobe medial (LLM) and lateral (LLL) segments in consensus. The ROIs were between 2-4 cm^2 ^in size. Care was taken to exclude vessels and motion artifacts from the ROIs. For each ADC value measurement, we applied three ROIs measurements to each segment and accepted the average.

Average of mean ADC values of the RLA and RLP segments was calculated as ADC-II. Mean ADCs, US, ECHO and blood biochemical parameters of both groups were compared.

## Statistical Analysis

NCSS 2007 & PAS 2008 Statistical Software (Utah, USA) were used for statistical analysis. Complementary statistical methods (mean, standard deviation) as well as student t-test was used in the comparison of quantitative data and parameters with normal distribution. Pearson correlation analysis was used to evaluate the relationship between parameters. Results were evaluated with a 95% confidence interval and p < 0.05 level of significance.

## Results

Etiological distribution of patients with PHT included 21 cases with rheumatic valve disease, 8 cases with left heart failure and 6 cases with chronic obstructive pulmonary disease. There was no difference between the PHT and control groups in terms of distribution of age and sex (p > 0.05).

Levels of LDH (232,60 ± 68,89 vs 182,11 ± 42,59; p = 0.002) and ALP (101,54 ± 45,18 vs 81,07 ± 21,90; p = 0.038) of the PHT group were higher than those of the control group.

Doppler echocardiographic evaluation of the PHT and control groups showed that mean PASP was lower in patients with normal RA compared to those with enlarged RA (p < 0.05). There was no difference in mean PASP in terms of RV and left atrial (LA) diameters (p > 0.05). EF was markedly lower in the study group compared to the control group (55,31 ± 9,51 vs 60,34 ± 4,56; p < 0.009).

### Ultrasonographic evaluation

Significant positive correlation and significant association was determined between liver size and diameter of IVC (43.1%), diameter of RA (38.3%), diameter of RHV (41.8%), diameter of MHV (40.5%) and LHV (41.1%) (Table 2).

**Table 2:** Correlation of liver size and related parameters

	Liver size	
	
	r	*p*
**Diameter of IVC (mm)**	0.431	0.001**

**LA(mm)**	0.184	*0.290*

**RA(mm)**	0.383	0.023*

**RV(mm)**	0.310	*0.070*

**EF(%)**	0.078	*0.657*

**ADC**	-0.014	*0.938*

**Diameter of VP(mm)**	0.079	*0.652*

**RHV(mm)**	0.418	0.013*

**MHV(mm)**	0.405	0.016*

**LHV(mm)**	0.411	0.014*

r: Pearson Correlation test * p < 0.05 ** p < 0.01

*Abb*: *ADC: *Apparent diffusion coefficient, *EF*: Ejection fraction, *IVC*: Inferior vena cava, *LA*: Left atrium, *LHV*: Left hepatic vein, *MHV*: Middle hepatic vein, *RA*: Right atrium, *RHV*: Right hepatic vein, *RV*:Right ventricle,*VP*: Vena porta.

There was no difference in terms of liver size, diameters of VP, IVC, RHV, MHV and LHV between patients with mild PHT and control group (p > 0.05). Mean ADC was higher in patients with moderate PHT compared to those with mild group (p = 0.025) (Table 3). Mean ADC and ADC-II were higer in patients with moderate PHT compared to those with control group (p < 0.01). Liver size was significantly greater among patients with moderate PHT compared to the control group (p = 0.008). There was no difference in terms of the diameters of IVC, VP, RHV, MHV and LHV among subjects with moderate PHT and the control group (p > 0.05) (Table 4).

**Table 3:** Evaluation of patients with mild PHT and the moderate group in terms of related parameters

	Mild PHT	Moderate PHT	
		
	Mean ± SD	Mean ± SD	
**Liver size(mm)**	141.45 ± 11.34	150.38 ± 13.73	***0.073***

**VP (mm)**	9.96 ± 1.79	9.37 ± 2.04	***0.436***

**IVC (mm)**	15.14 ± 3.66	15.37 ± 4.14	***0.882***

**RHV(mm)**	7.08 ± 2.01	8.62 ± 3.93	***0.317***

**MHV(mm)**	7.14 ± 2.16	8.95 ± 3.21	***0.075***

**LHV(mm)**	7.09 ± 2.04	8.08 ± 2.83	***0.280***

**ADC(mm**^**2**^**/s)**	1.62 ± 0.22	1.82 ± 0.16	0.025*

**ADC-II(mm**^**2**^**/s)**	1.56 ± 0.18	1.72 ± 0.19	0.025*

Students t test * *p < 0.05*

Abb*: ADC*:Apparent diffusion coefficient, *IVC*:Inferior vena cava, *LHV*:Left hepatic vein, *MHV*:Middle hepatic vein, *PHT*:Pulmonary arterial hypertension, *RHV*:Right hepatic vein, *VP*:Vena porta.

**Table 4:** Evaluation of patients with moderate PHT and the control group in terms of related parameters

	Moderate PHT	Controls	*p*
		
	Mean ± SD	Mean ± SD	
**Liver size(mm)**	150.38 ± 13.73	139.28 ± 8.25	0.008**

**VP(mm)**	10.08 ± 1,84	9.37 ± 2.04	0.362

**IVC(mm)**	15.37 ± 4.14	14.09 ± 2.56	0.295

**RHV(mm)**	8.62 ± 3.93	7.11 ± 1.95	0.325

**MHV(mm)**	8.95 ± 3.21	6.92 ± 1.31	0.122

**LHV(mm)**	8.08 ± 2.83	6.87 ± 1.34	0.276

**ADC(mm**^**2**^**/s)**	1.82 ± 0.16	1.62 ± 0.17	0.009**

**ADC-II(mm**^**2**^**/s)**	1.73 ± 0.19	1.55 ± 0.18	0.025*

Students t test * p < 0.05 ** *p < 0.01*

Abb*: ADC*:Apparent diffusion coefficient, *IVC*:Inferior vena cava, *LHV*:Left hepatic vein, *MHV*:Middle hepatic vein, *PHT*:Pulmonary arterial hypertension, *RHV*:Right hepatic vein, *VP*:Vena porta.

### ADC measurements with DW-MRI

**Figure 1:**
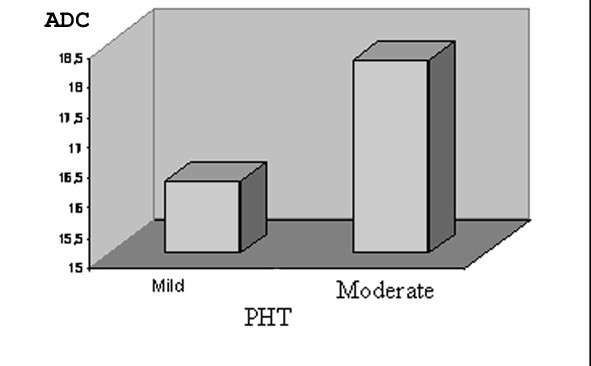
**Bar graphic of the distribution of mean ADC by the degree of PHT**. *Abb: ADC*; Apparent diffusion coefficient, *PHT*; Pulmonary arterial hypertension.

**Figure 2:**
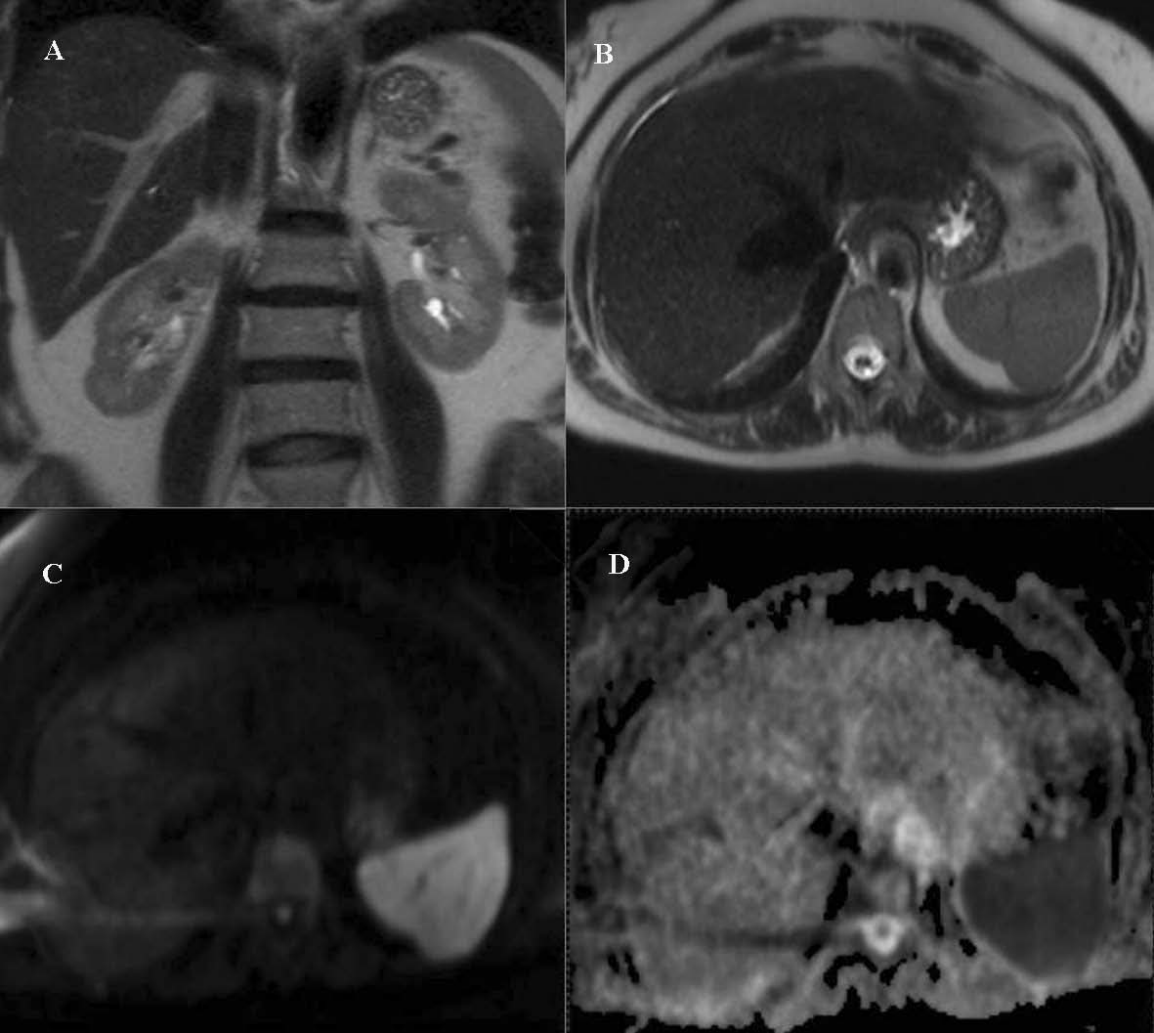
**MRI sequences of a patient with moderate PHT (43 year old female).** There is slight enlargement of IVC and hepatic veins. **A **Coronal T2-weighted sequence, **B **Axial T2-weighted sequence **C **Axial diffusion-weighted image (b = 1000 s/mm^2^), **D **ADC map of the same patient; calculated ADC was 1.86 × 10ˉ³ mm²/s (increased diffusion due to intercellular fluid accumulation that is not apparent visually, however detectable with ADC calculation).

Mean ADC (1.82 ± 0.16) was higher in patients with moderate PHT compared to those with mild PHT (1.62 ± 0.22) (p = 0.025) (Figure 1). While mean ADC and ADC-II were higher in patients with moderate PHT compared to the control group (p < 0.05) (Table 4), mean ADC-II was not different in patients with mild PHT and control group (p = 0.297). The DW-MR images of a moderate PHT patient are shown in Figure 2.

## Discussion

PHT may lead to chronic passive hepatic congestion and ischemic hepatic dysfunction due to secondary right ventricular dysfunction. Increased right heart chamber pressures secondary to physiopathological changes, in pulmonary arteries result in dilatation of IVC and hepatic veins. Stasis in the hepatic sinusoids stem from elevated hepatic vein pressure cause hepatocellular hypoxia and necrosis. These hepatic changes may present clinically as hepatomegaly, ascites, edema and subicterus. Serum bilirubin, transaminase, ALP and prothrombin times may be increased as a result of hepatic dysfunction [14-18]. In our patients we determined increased indirect bilirubin, ALP and particularly LDH due to ischemic effects of congestion. Increased AST, LDH and indirect bilirubin often presents with cardiac pathologies (30-50%) [19-21]. In the study of Lau *et al*., levels of ALP, ALT, bilirubin and GGT were significantly increased in conformity with the level of tricuspid failure [12]. Elevated levels of ALP, LDH and indirect bilirubin are important findings supporting the importance of biochemical markers in demonstrating hepatic congestion.

Sensitivity and specificity of PASP measurement with Doppler echocardiography compared to right-heart catheterization are 100% and 96%, respectively [5,6].

Several studies, including that of Jayant Nath *et al*. have demonstrated that mortality rates increase in the presence of dilatation and dysfunction of right heart chambers [22-25]. We also determined significant relationship between RV and RA size and PASP (p = 0.038, p = 0.040, respectively).

Although used commonly in clinical practice, the sensitivity and specificity of US varies in different liver pathologies (passive congestion, hepatitis, mass lesions, fibrosis etc.). A positive correlation was determined between liver size and diameters of IVC, RA, RHV, MHV and LHV (Table 4); however, liver size was enlarged compared to the control group only in subjects with moderate PHT. Even though it was not statistically significant, VP, IVC, RVH, MHV and LHV values of the moderate PHT group were increased when compared with mild PHT and control groups. This may be a consequence of the relatively low number of the study group. There was no difference between subjects with mild PHT and control group in terms of liver size and diameters of VP, IVC, RHV, MHV and LHV (p < 0.05). This indicates that no morphological changes are determined with US in mild PHT, whereas in moderate PHT the only change is increased liver size which is better diagnosed in moderate-severe PHT.

Diffusion-weighted imaging is a new imaging modality recently implemented to be used in abdominal diseases including diffuse liver diseases. This modality takes approximately 3 minutes in addition to a routine abdominal MRI and is a non-invasive procedure, which does not require contrast material injection. Its advantages include application without breath holding, repeatability and relative cheapness. This modality may also be used in the follow up of patients by making quantitative measurements on ADC map constituted from diffusion images [26-29]. Diffusion includes movement behaviors of molecules in microscopic random pattern and this movement is measured from mean diffusion coefficient. DW-MRI is sensitive to this movement that is measured with ADC, and water diffusion is measured with ADC [30]. ADC is often higher than anticipated in biological tissues due to the fact that the term microscopic movement includes molecular diffusion of the water and microcirculation of blood in capillary network. In other words, ADC is influenced by both diffusion and perfusion. MRI diffusion measurements might be influenced by several factors including perfusion, cellular structure and permeability [31,32].

Accurate detection and characterization of focal and diffuse hepatic lesions can be performed with MR imaging. Various sequences, such as inversion recovery, gradient echo, spin echo seqıences are highly sensitive for the detection of lesions. Once detected, they can often be accurately characterized as malignant or benign, cyst or solid tumor, etc., based upon their appearance and relative signal intensity on T1- and T2- weighted sequences with the help of fat supression techniques and intravenous contrast material administration. Diffuse liver diseases like fatty infiltration, iron accumulation or fibrosis may be detected with these sequences [33]. However, because of the characteristics of gradual development of the diseases and no obvious morphological changes, conventional imaging which only illustrates anatomical configurations can not provide valuable information for clinical diagnosis especially in the initial phases. Neither the hoarse echo of ultrasound nor minor signal alteration on T2 weighted imaging of MRI could provide quantitative criteria. DW-MRI, however, can detect the changes of tissue structure at molecular level. Quantitative DW-MRI ADC measurement, is sensitive to detect hepatic diffuse lesions in the early stage [34,35]. Several studies have shown that the ADC of cirrhotic liver is lower than that of normal liver [36]. ADC measurements are potentially useful for the evaluation of fibrosis staging in the liver [37-39].

DW-MRI studies aimed at demonstrating liver diseases have shown limited diffusion and decreased ADC value particularly in hepatitis, liver fibrosis and cirrhosis due to reduced intercellular water and fibrotic reactions [26-28]. Reduction in DW-MRI accompanied with reduction in ADC by the damage in diffuse parenchymal liver disease [40] may indicate the possible benefit in differential diagnosis of congestion. Contrary to the literature, however, we found increased intercellular water and diffusion and thus increased ADC accompanying passive stasis. Our study is the first study performed on passive congestion and our results are compatible with basic diffusion knowledge [30-32].

Clinically ultrasonography is used in the evaluation of liver parenchyma and is capable of showing morphological changes resulting from passive congestion particularly in cases with PHT. In our study, changes in liver parenchyma have been demonstrated in patients with moderate PHT using DW-MRI. LDH (p = 0.002) and ALP (p = 0.038) levels of patients with PHT were determined to increase compared with control patients. Although LDH and ALP increase may be noted in several liver pathologies other than hepatic congestion, mean ADC is decreased rather than increased in fibrotic liver parenchymal diseases [32,41]. The facts that mean ADC was higher in patients with moderate PASP compared to mild PASP and that mean ADC plus ADC-II were elevated in patients with moderate PHT confirm that the perfusion effect resulting from cardiac effect on the liver may be demonstrated on DW-MRI. Several previous studies have thus suggested that posterior right liver lobe should be preferred over left liver lobe in ADC measurements [42,43]. Therefore, our right lobe ADC-II measurement was found to be higher than that of the control group. This finding suggests that liver congestion is primarily localized in RLA and RLP segments which may be shown with DW-MRI.

The therapeutic options for PHT have significantly improved in the last few years. But the disease is still too often diagnosed late in its course. In more than 80% of patients the diagnosis is made and treatment started when the condition is already in NYHA class III-IV with a very poor prognosis. According to the EARLY study, beginning treatment 3-6 months earlier can improve prognosis and progression of the PHT [44]. Late diagnosis is first of all due to the fact that it is asymptomatic in the early stages, such symptoms as dyspnoea, peripheral edema or syncope frequently occurring only when there are already signs of heart failure. Secondly, there is as yet no method for the early diagnosis of PHT. But echocardiography and cardiac MRI are non-invasive modes that are important for screening and follow-up. Among noninvasive methods echocardiography shows the greatest diagnostic sensitivity and specificity. However this method has been considered in frequently symptomatic patients with clinical progression and poor prognosis of the disease [45,46].

In our study, the congestion related alterations in USG and DW-MRI investigations were also evident among patients with PTH without symptom and clinically overt heart failure. Identification of significant differences in RLA and RLP in congested liver seems to be the superiority of DW-MRI to USG in evaluation of cardiac congestion. Additionally DW-MRI was shown in our study as a different radiological method enabling detection of cardiac congestion before the development of heart failure. The efficacy of early treatment induction (such as diuretics) in patients with PTH has been a well-known phenomenon as demonstrated by past studies [44]. This may indicate that the degree of liver congestion and treatment response could be evaluated by DW-MRI as a non-invasive method enabling differential diganosis of hepatomegaly and also cardiac congestion dependent hepatomegaly as well as ealier induction of treatment in PTH.

The limitations of the study: MR is not often available and it is not that cheap. Moreover, these patients are often orthopneic and it may be difficult to perform an MR. However technological advances in the field may overcome at least some of these problems by shortening the examination duration.

Consequently, DW-MRI performed to diagnose hepatic congestion secondary to moderate PHT is an easy-to-use, non-invasive and repeatable test that gives quantitative results and may be used in conjunction with routine MRI modalities. In addition, hepatic congestion (with increased ADC) may be easily distinguished from diffuse parenchymal liver diseases characterized with reduced ADC values. Collectively, these data indicate that further studies should be performed to clarify the place of this modality in both cardiac and other congestive liver pathologies.

## Competing interests

The authors declare that they have no competing interests.

## Authors' contributions

YD, AS, OK, TD, KOK collected all data and participated in the design of the study. YD and OK were supervisor of the echocardiographic, and MRI examinations.

SND, GAE, NY, SP and KS drafted the manuscript with YS. All authors read and approved the final manuscript.
